# Ulceration of the Tongue Cured by the Removal of Two Amalgam Fillings from the Teeth

**Published:** 1851-04

**Authors:** 


*Ulceration of the Tongue Cured by the Removal of Two Amalgam Fillings from the teeth.*—Mrs. ——, a lady of high respectability of Baltimore, about twenty-eight years of age, called on us, the 17th of May, 1850, to obtain our opinion with regard to the condition of her teeth, and to have such operations performed on them as they might require. On examining her mouth, our attention was attracted to an ulcer, of a somewhat indurated character, on the under part of the right side of her tongue, where it came in contact with the grinding surface of the last molar tooth. Observing a large amalgam filling in this tooth, with a black oxydized surface, though it had apparently been put in and finished in a very perfect manner; it immediately occurred to us that the ulcer of the tongue might be connected, in some way, with it—or rather, that it had been caused by the action of a corrosive oxyd, formed by the union of the mercury with some of the acids of the mouth. The correctness of this opinion seemed to be confirmed by the fact, which we learned from the lady, that the ulcer had not developed itself until some two or three months subsequently to the introduction of the amalgam into the tooth, which had then been in about nine months. Although it had been touched several times with nitrate of silver, it manifested no disposition to heal; on the contrary, it had for some time been gradually assuming a more and more aggravated character. On mentioning our suspicions, the lady at once determined to have the tooth removed, as she had been assured it could not be filled with any other material. We, however, dissuaded her from this, advising that the amalgam should be first removed, and then, if the tooth could not be filled with gold, we told her it would be time enough to extract it.

After taking the amalgam from the tooth, we proceeded to fill it with gold, four hours and a half being occupied in the operation.

The ulcer in the tongue almost immediately began to assume a healthier character, and entirely disappeared in about six weeks.

About this time we were requested to perform any other operation upon the lady's teeth which might be required for their preservation, and as the socket of the second molar on the other side of the mouth, in the lower jaw, was the seat of a very troublesome abscess, we at once removed the tooth. This exposed the surface of another large amalgam filling, in the anterior approximal surface of the third molar, and with which the tongue occasionally came in contact. The first and third molars having become somewhat loosened from inflammation of the peridental membrane, caused by disease in the socket of the second, we concluded to defer operating on these teeth until the adjacent parts should have assumed a healthy condition. In the mean time, some three or four weeks after the extraction of the above mentioned tooth, an ulcer developed itself on the side of the tongue, just where it came in contact with the amalgam filling in the third molar. A few weeks subsequently the amalgam was removed and the cavity filled with gold. The ulcer soon after healed.

Now what connection the amalgam fillings had with the ulcers on the sides of the tongue in this case, we leave others to determine, contenting ourself with simply mentioning the facts of the case. Comments are unnecessary.

